# Deep neural network-based phase calibration in integrated optical phased arrays

**DOI:** 10.1038/s41598-023-47004-z

**Published:** 2023-11-15

**Authors:** Jae-Yong Kim, Junhyeong Kim, Jinhyeong Yoon, Seokjin Hong, Berkay Neseli, Namhyun Kwon, Jong-Bum You, Hyeonho Yoon, Hyo-Hoon Park, Hamza Kurt

**Affiliations:** 1 School of Electrical Engineering, Korea Advanced Institute of Science and Technology (KAIST), Daejeon, 34141 Republic of Korea; 2 National Nanofab Center (NNFC), Daejeon, 34141 Republic of Korea

**Keywords:** Integrated optics, Photonic devices

## Abstract

Calibrating the phase in integrated optical phased arrays (OPAs) is a crucial procedure for addressing phase errors and achieving the desired beamforming results. In this paper, we introduce a novel phase calibration methodology based on a deep neural network (DNN) architecture to enhance beamforming in integrated OPAs. Our methodology focuses on precise phase control, individually tailored to each of the 64 OPA channels, incorporating electro-optic phase shifters. To effectively handle the inherent complexity arising from the numerous voltage set combinations required for phase control across the 64 channels, we employ a tandem network architecture, further optimizing it through selective data sorting and hyperparameter tuning. To validate the effectiveness of the trained DNN model, we compared its performance with 20 reference beams obtained through the hill climbing algorithm. Despite an average intensity reduction of 0.84 dB in the peak values of the beams compared to the reference beams, our experimental results demonstrate substantial agreements between the DNN-predicted beams and the reference beams, accompanied by a slight decrease of 0.06 dB in the side-mode-suppression-ratio. These results underscore the practical effectiveness of the DNN model in OPA beamforming, highlighting its potential in scenarios that necessitate the intelligent and time-efficient calibration of multiple beams.

## Introduction

Integrated optical phased arrays (OPAs) have emerged as a crucial technology for enabling free-space beam forming and steering in various applications, including light detection and ranging (LiDAR), optical wireless communication, virtual reality (VR), augmented reality (AR), and bio-sensing^1–7^. In particular, silicon-on-insulator (SOI) platform-based OPAs have been extensively studied due to their high index contrast, providing high integration, compactness, and compatibility with complementary metal-oxide-semiconductor (CMOS) technology^8–15^. These inherent advantages have led to the development of Si-based integrated OPAs with over 1000 channels^16^, specifically designed to achieve a small beam divergence angle, making them highly advantageous for LiDAR applications. However, as the number of channels in Si-based OPAs scales up, ensuring precise phase control for each channel becomes increasingly challenging. The presence of crosstalk (both electrical and thermal drifts) between arrayed channels and fabrication errors can no longer be negligible in densely integrated areas with a large number of channels^17^. These factors contribute significantly to phase errors, requiring the phase calibration for each channel in order to achieve precise beamforming during beam steering.

Conventional approaches for phase calibration in OPAs have relied on optimization-based methods such as the hill-climbing algorithm (HA)^18^, genetic algorithm (GA)^19^, and particle swarm optimization (PSO)^20^. These iterative and optimization-based calibration processes can effectively align the wavefront and generate desired beam-forming outcomes. However, it is crucial to acknowledge that reliance on these optimization methods requires significant computational time, especially for the OPAs with increased channels. For example, the HA is difficult to implement for a large-scale OPA as it exhaustively searches the optimum electrical signals in an iterative manner. GA or PSO can be deployed to make phase calibration of large-scale OPA. However, such methods have the main drawback of long convergence time and they may get stuck in the local optimum. With the advancing complexity and functionality of OPAs, there is a strong demand for an enhanced beamforming method, which can provide an accurate and efficient phase calibration process.

Recently, the emergence of artificial neural networks (ANNs) has introduced innovative solutions across various fields in nanophotonics^21–23^. Specifically, successful implementations with high performances were achieved in two-photon microscopy^24^, optical metrology^25^, inverse designing nanophotonic devices^26–31^, resolving complex ellipsometry problems^32^, and photonic design correction^33^. With their capability to handle complex nonlinear mappings, ANNs present an ideal foundation for developing a framework to address OPA calibration and there have been some notable advancements in utilizing ANN techniques for OPA beamforming. Researchers have explored the application of an ambiguity-resolved fully-connected layer to achieve OPA phase calibration for a single beam point with a high side-mode-suppression ratio (SMSR)^34^. Additionally, a convolutional neural network (CNN) has been employed to predict the required current set for phase control, enabling successful 2-D beam steering with multiple beam points^35^. Although these studies highlight the potential of ANN-based approaches in enhancing the calibration and beamforming capabilities of OPAs, they were primarily devoted to OPAs with smaller channels, typically 16 or fewer. There has been research on DNN-based phase control for 128-channel OPA beamforming as well^36^. However, it necessitated a substantial dataset of 350,000 samples for training with training epochs of 2500. Therefore, further research is required to evaluate the performance and scalability of these ANN-based methods when applied to OPAs with a larger number of channels, with an emphasis on efficient approaches for training the network.

In this paper, we introduce a DNN-based phase calibration method to enhance beamforming in a 64-channel optical phased array (OPA). To effectively handle the complexity of voltage control, we employ a tandem network architecture^26,29,31^, and further enhance its performance through selective data sorting and hyperparameter tuning. For evaluation, we utilize 20 beams generated by the HA algorithm as evaluation datasets and fed them into the trained network. The experimental results indicate a slight intensity degradation of 0.84 dB and a minor decrease of 0.06 dB in the side-mode-suppression-ratio (SMSR) compared to reference beams. Nonetheless, they clearly demonstrate a substantial alignment between the DNN-predicted and reference beams, underscoring the practicality of the DNN model for intelligent and efficient calibration in a variety of scenarios.

## Device design and fabrication

**Figure 1:**
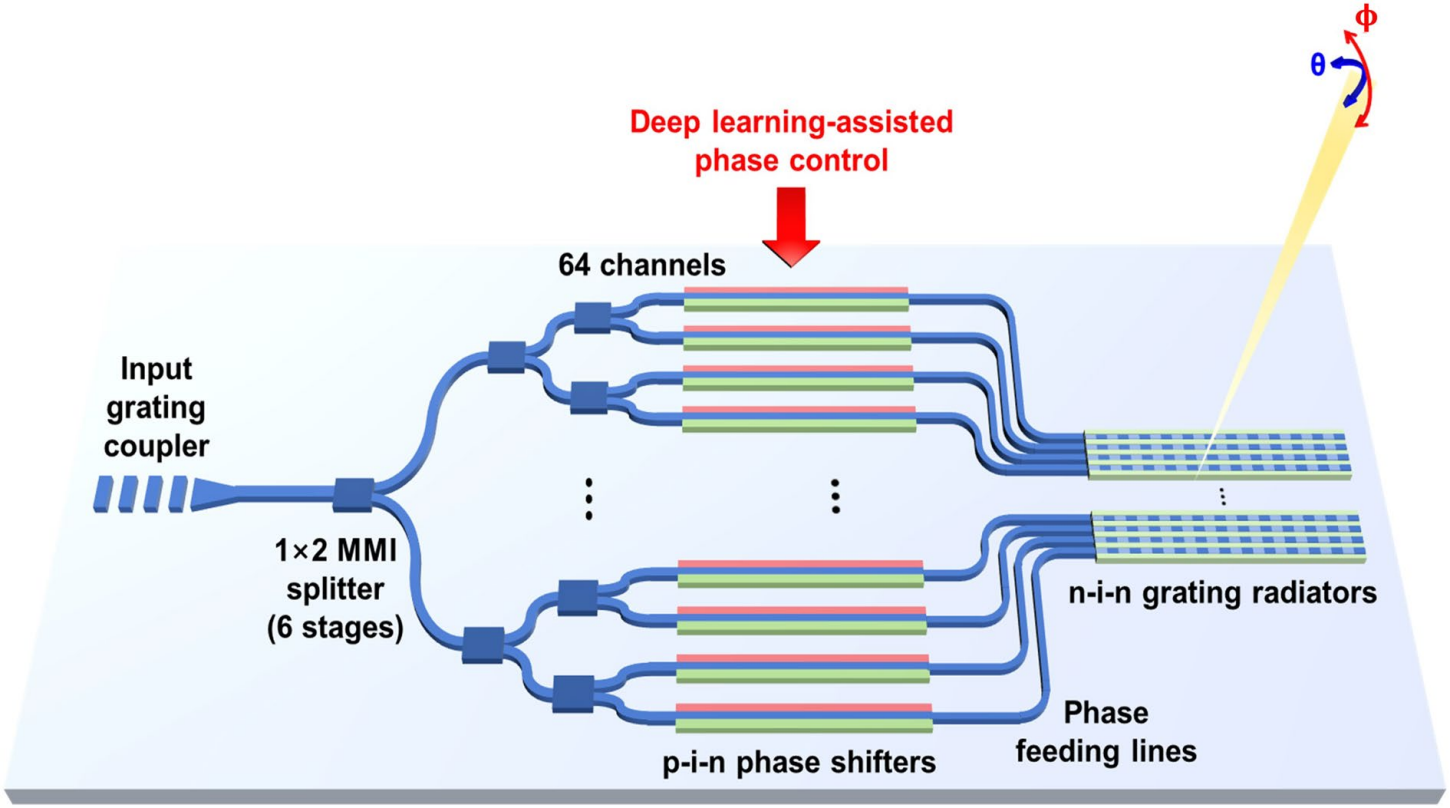
Schematic of 64-channel integrated optical phased array (OPA).

The schematic of the 64-channel integrated silicon-based OPA chip is illustrated in Fig. 1. The designed OPA chip consists of an input grating coupler, 6-stage multimode interferometer (MMI) splitters, 64 electro-optic (EO) phase shifters, phase feeding lines, and grating-based radiators with an n-i-n structure. These components are specifically designed for transverse electric (TE) polarization mode propagation. To begin, the incident light is coupled into a silicon waveguide using the input grating coupler and is then evenly distributed into a 64-channel waveguide through a series of MMI splitters. Each of the 64 channels is integrated with phase shifters having a p-i-n junction structure, allowing control of the refractive index based on the electro-optic (EO) effect. The phase-tuned light in each channel is then guided through the pan-in-shaped phase-feeding lines, connecting the phase shifters to the grating radiators. Finally, the light is emitted through the grating radiator array, in accordance with the same specifications as our previous work^3^, which features a 300 $\upmu$m length of uniform grating structure with a 20 nm etch depth, a period of 590 nm on a waveguide width of 1 $\upmu$m. The grating radiator array was designed to have a uniform pitch of 2 $\upmu$m, providing the capability of transversal beam-steering within a range of 45$^\circ$. To enable beamforming and beam steering in the transversal direction, the p-i-n phase shifters in each channel provide the capability to manipulate the phase of the emitted wavefront from the grating radiator array and this manipulation is accomplished by applying a forward-biased voltage to control the refractive index^37^. In addition, thermo-optic (TO)-based tunable grating radiators were applied for longitudinal beam-steering, employing the n-i-n heaters^3, 38^. Applying currents to the heaters, the effective refractive index of the grating region is changed by the TO effect and the beam can be longitudinally steered within a range of 10$^\circ$ with a tuning efficiency of 0.67$^\circ$/W^38^. However, we should note that this study primarily concentrates on the phase control aspects related to transversal beam steering, and the details of longitudinal beam steering using thermo-optic tuning are not discussed in-depth.

In our proposed method, we primarily focus on addressing the challenges associated with controlling transversal beam steering, which can be more challenging due to the multi-channel configuration and the presence of active control for the phase shifters. For a linear, uniform pitched *N*-channel OPA operating at an input wavelength, the theoretical far-field beam intensity distribution I($\theta$, $\phi$) can be expressed as follows:

1 $$\begin{aligned} I(\theta , \phi )=g(\theta , \phi )\left| \sum _{m=1}^N A_{m}\left\{ -j\left[ k_{0}d\sin (\theta )+\varphi _{m} \right] \right\} \right| \end{aligned}$$

where *g*($\theta$, $\phi$) is the far-field envelope created by a single radiator, $A_{m}$ is the element factor of the $m_{th}$ radiator, $k_{0}$ is the wave number of the incident light in vacuum, *d* is the pitch of the radiator array, $\theta$ and $\phi$ are the transversal and longitudinal angles respectively and $\varphi _{m}$ is accumulated phase at the output of the $m_{th}$ channel. To achieve the desired far-field beam pattern *I*($\theta$, $\phi$), precise control of the optical phase $\varphi _{m}$ is required for accurate beamforming in the desired direction. In practical environments, however, achieving precise control of the optical phase $\varphi _{m}$ is challenging due to the susceptibility to various phase errors introduced by inevitable factors such as fabrication errors, crosstalk between adjacent channels, and electrical or thermal drift. These factors add to the nonlinearity of the phase control and contribute to increasingly severe phase errors with the scaling of the OPA, resulting in unpredictable and random phase errors. Moreover, the implementation of phase control through the EO phase shifters introduces optical loss variations in each channel. These variations are dependent on the amount of current injected into them and also hold the potential to impact the output far-field beam intensity distribution^37^. Therefore, it is crucial to implement independent optical phase control to mitigate these unpredictable phase errors and amplitude variations in each OPA channel. Our approach, employing deep learning-based phase control as depicted in Fig. 1, aims to provide a solution to facilitate the calibration process for beam steering with distinct advantages over conventional optimization-based methods.

**Figure 2:**
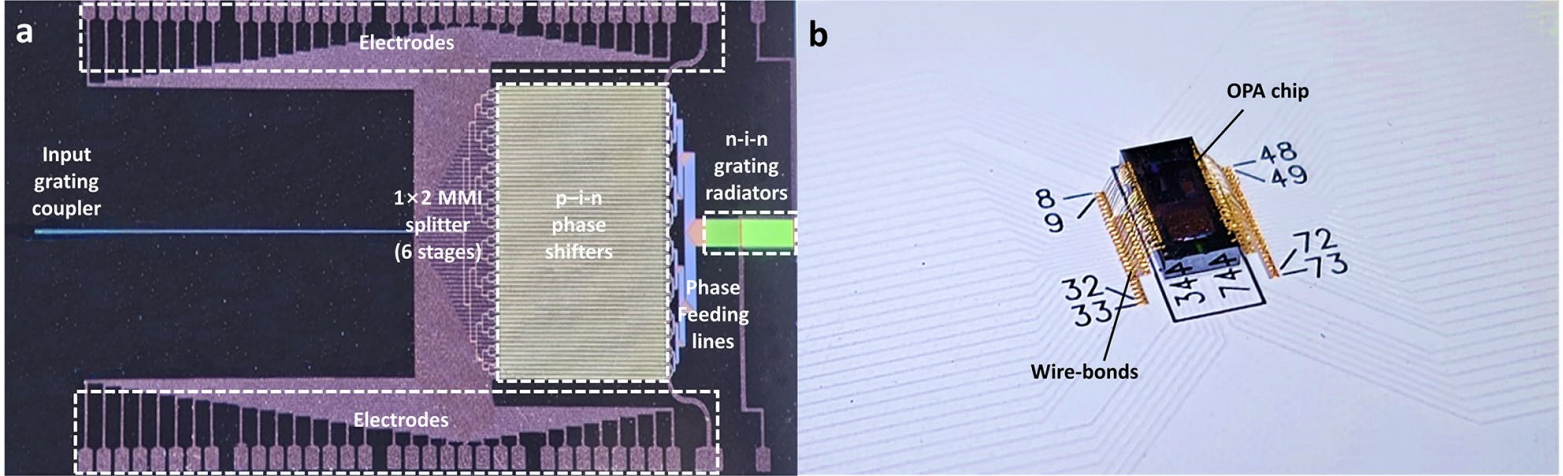
(**a**) Fabricated 64-channel OPA chip and (**b**) electrical wire-bonding packaged OPA chip on printed circuit board (PCB).

The OPA chip was manufactured in the National Nanofabrication Center (NNFC) using a CMOS-compatible fabrication process on an 8-inch silicon-on-insulator (SOI) wafer with a top silicon layer thickness of 220 nm and a buried oxide layer thickness of 2 $\upmu$m. A KrF scanner with a 248 nm wavelength laser was utilized in the lithography process. The overall footprint of the fabricated chip including the electrode pad shown in Fig. 2a, was 5 mm $\times$ 2.2 mm. Note that the end-to-end optical loss of our fabricated OPA sample was estimated to be approximately 25.2 dB. This comprehensive loss can be attributed to specific components as follows: 6.5 dB from the coupling losses at the input grating coupler, 3.2 dB from the 6-stage MMI splitters, 4.5 dB from the p-i-n phase shifter, and 11 dB from the n-i-n grating radiator. The average power consumption for achieving a $2\pi$ phase shift in the 64-channel phase shifters was measured to be 12.2 mW. To facilitate the voltage control for precise phase tuning of the OPA chip, a metal printed circuit board (PCB) was additionally designed and manufactured to accommodate the overall size of the OPA chip. The electrode pads in the OPA chip were then wire-bonded to the fabricated PCB, as depicted in Fig. 2b.

## Experimental setup and data preparation process

**Figure 3:**
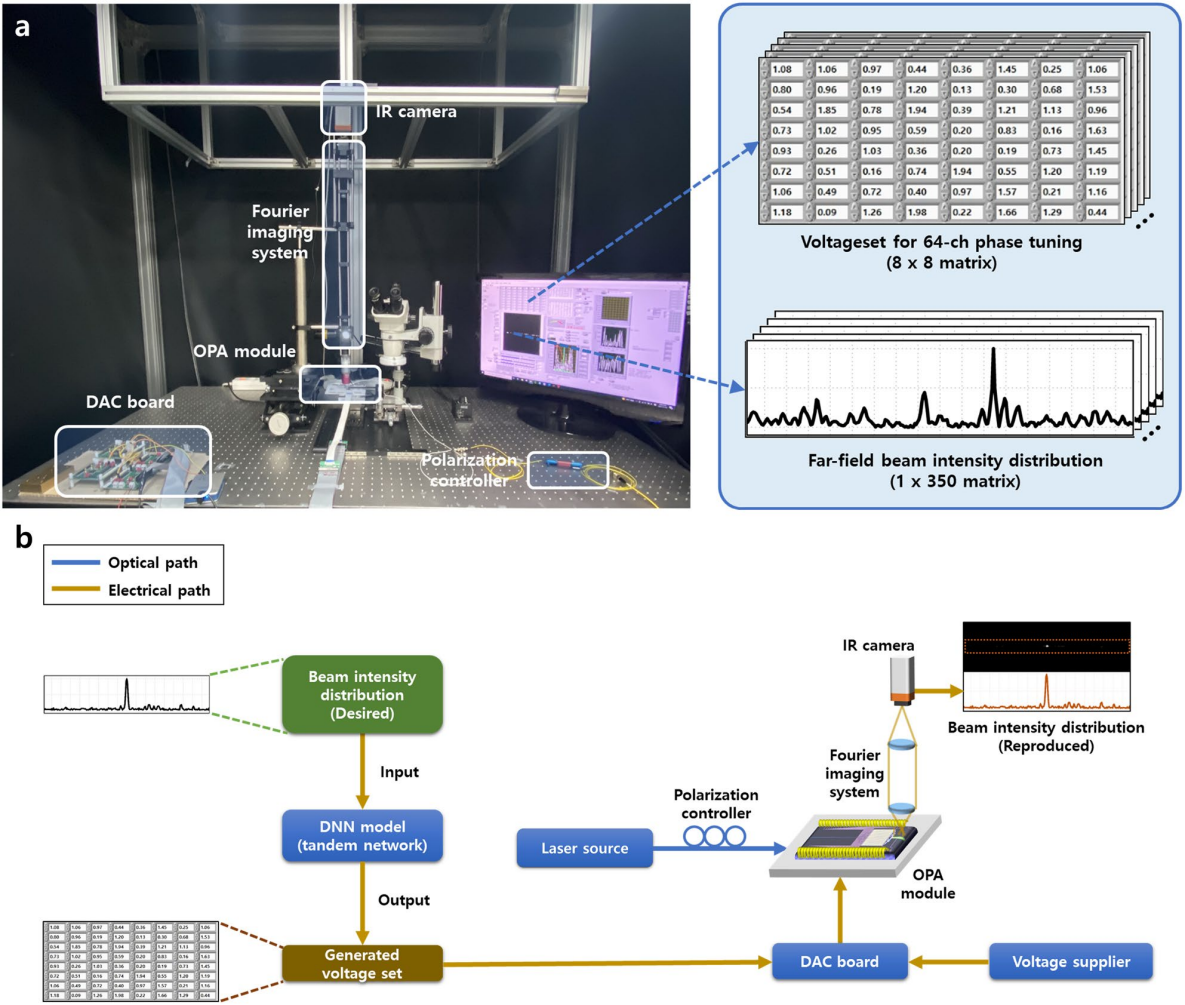
(**a**) Experimental setup for gathering the dataset to train the DNN model for OPA beamforming. The setup generates a voltage dataset for 64-channel phase tuning ($8 \times 8$ matrix) and the corresponding far-field beam intensity distribution dataset ($1\times 350$ matrix). (**b**) Schematic diagram illustrating the overall experimental setup for reproducing the desired beam intensity distribution using DNN model-assisted phase control.

Figure 3a shows the experimental setup to gather the dataset for training and evaluating the phase control capability of the neural network model. The 1550 nm optical signal from the laser source was coupled into the OPA module through a grating coupler via a polarization controller to maintain the TE polarization mode. To minimize measurement errors, fiber packaging with UV-cured epoxy was pre-performed on the input grating coupler, ensuring enhanced stability and consistent results. To capture the far-field beam distribution from the OPA radiators, a Fourier imaging setup was employed, comprising an infrared (IR) camera, a two-tube lens, and a microscope objective with a numerical aperture (NA) of 0.65. This configuration enables the coverage of a measurement range over ± 20$^\circ$ from the center in the far-field image. From the resulting 2-D far-field beam image, the corresponding 1-D beam distribution was also extracted. To control the driving voltage applied to the 64 electrodes on the OPA module, a digital-to-analog converter (DAC) board was utilized, which was connected to the PC and controlled by our custom LabVIEW software (version 17.0f2)^39^, allowing precise control over the voltage applied to each electrode. These extracted data from the setup are used to train our DNN model. After the training process, the trained model is then implemented to reproduce the beam intensity distribution in the desired direction by inputting the generated voltage set to the OPA, as depicted in Fig. 3b.

In order to train our model for OPA phase control using a data-driven approach, a substantial number of samples were required from this experimental setup. These samples consisted of a voltage dataset for 64-channel phase tuning ($8\times 8$ matrix) and the corresponding far-field beam intensity distribution dataset ($1\times 350$ matrix), as depicted in Fig. 3a. This matrix size of 350 elements in the far-field beam intensity distribution dataset was chosen to extract data from a transversal range of 50$^\circ$, with each pixel representing an angular resolution of 0.14$^\circ$. The range of voltage applied to the 64 phase shifters was limited from 0.6 to 1.6 V, ensuring sufficient $2\pi$ phase shifts for each channel. So, if we were to consider all possible cases with a voltage tuning increment of 0.01 V, there would be a total of  $1.9\times 10^{128}$ possible combinations. However, due to limitations in time and computational resources, it was impractical to gather datasets for all possible combinations for training the model. Hence, our approach was to selectively extract the meaningful samples and train our model to attain the prediction performance that closely matches that of conventional optimization-based algorithms.

For preparing the training and testing samples, we initially generated a total of 90,000 voltage sets for the index tuning of 64 phase shifters. Each voltage set consisted of 64 random variables following a uniform distribution ranging from 0.6 to 1.6 V with increments of 0.01 V. A minimum voltage of 0.6 V was chosen, considering the operational threshold voltage range of 0.7–0.8 V for each p-i-n phase shifter. Beginning from 0.6 V instead of 0.0 V also provided the advantage of narrowing the data range, thereby facilitating the training of the network. For the maximum voltage of 1.6V, it was determined considering various factors leading to deviations across channels, such as channel-to-channel crosstalk and variations in metal resistance due to different routing in each channel. These factors resulted in voltage deviations of up to 0.2 V to achieve a $2\pi$ phase shift in each channel of the OPA sample. Consequently, we extended the maximum voltage to 1.6 V to accommodate these variations with a 0.1 V margin, even though only 1.3 V was required for a $2\pi$ phase shift in a single-phase shifter. In order to ensure stability and minimize the risk of program errors during the data collection process, we prudently selected a data collection interval of 400 ms, resulting in the accumulation of over 90,000 datasets in 10 h. We should note that there is room to minimize the data collection time, as the time is primarily determined by the speed of the DAC board and the IR camera’s frame rate, totaling approximately 10 ms in our experimental setup.

Here, it is important to note that our primary objective was to find voltage datasets that would yield peak intensity values in the desired beam direction. To achieve this, we selectively collected datasets where the maximum value of the beam intensity distribution exceeded a predefined threshold intensity, instead of utilizing all 90,000 datasets for training the model. This selective collection process resulted in a final dataset of 18,000 samples, each consisting of voltage datasets and their corresponding beam distributions. Among the samples, 80% were assigned as training samples, while the remaining 20% were labeled as test samples. Additionally, we also employed additional samples using the HA as the reference for evaluating the proposed model’s performance in OPA beamforming. Using this algorithm, we searched voltage sets for 20 different beam points aligned with desired beam directions within a transversal beam steering range of 45$^\circ$ for our OPA, and the corresponding far-field beam distributions were also obtained for these datasets. These datasets were then used later to assess and compare the performance of our trained neural network model against the results generated by the conventional hill-climbing algorithm.

## Design of the deep neural network model

**Figure 4:**
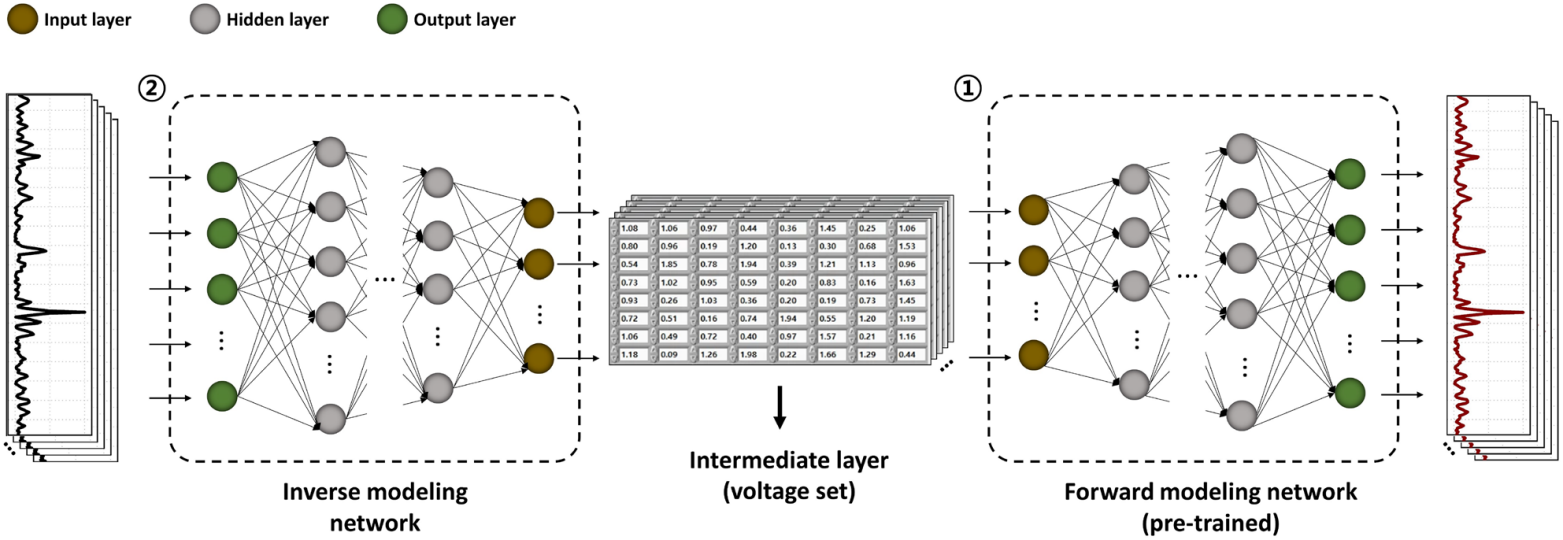
Schematic diagram of the proposed tandem network model used for the OPA phase control. The model comprises 2 interconnected networks: the forward modeling network (pre-trained) and the inverse modeling network. This tandem network effectively captures the complex input-output relationships required for precise and efficient beam steering in multiple OPA channels.

Given the utilization of the 64-channel OPA in our work, there is a broader range of possibilities for manipulating phase combinations to achieve one specific beam direction. However, this increased flexibility can also give rise to a non-uniqueness problem, similar to what is encountered in the inverse design of photonic structures when relying solely on an inverse modeling network^26^. To tackle this expected challenge, it is crucial to design a deep neural network that can effectively handle the complexity of the problem. Hence, as shown in Fig. 4, we employed a fully-connected layer-based tandem network architecture for the phase control of our OPA, which combines a pre-trained forward modeling network with an inverse modeling network. This proposed architecture can facilitate a one-to-one mapping between the desired beam distribution and the corresponding voltage set, effectively reducing the complexity of the training process in a deep neural network for scaled OPA phase control. Our network was implemented with the PyTorch 2.0 platform and trained using an NVIDIA GeForce RTX 3070 Ti with 64 GB RAM.

The detailed overall process is as follows. First, we trained the forward modeling network that connects the voltage set with the corresponding far-field beam distribution. Once the forward model was successfully trained, we then fixed its weights and trained an inverse modeling network that inversely connects the far-field beam distribution with the voltage set. This inverse modeling network was designed to reversely predict the voltage set from the desired far-field beam distribution. Then, we connected the pre-trained forward modeling network after the inverse modeling network, forming a tandem network as shown in Fig. 4. This allowed us to ensure a one-to-one mapping between the voltage set and the far-field beam distribution, ensuring the consistency and reliability of the network. Additionally, we employed the extracted voltage set from the intermediate layer of the tandem network for phase control in the OPA beam forming.

**Figure 5:**
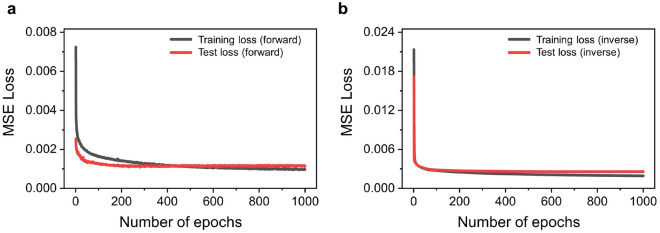
The learning curves of (**a**) the forward modeling network and (**b**) the inverse modeling network.

Our initial focus was on training the forward modeling network, which establishes the connection between the voltage set and the corresponding far-field beam distribution. Prior to the training process, we performed normalization on each far-field beam intensity distribution by dividing it by the maximum intensity value among all data samples. This step was implemented to minimize the loss function and enhance the training procedure. We used a Mean Square Error (MSE) for the loss function to minimize the overall deviation between the predicted values and the actual values during the training process. In addition, we employed the Optuna library^40^, which provides a hyperparameter tuning method, to optimize the weights of the forward model. This process involved optimizing various hyperparameters, such as the number of hidden layers, the number of nodes in each hidden layer, dropout values between the hidden layers, and learning rates to minimize a training loss and a testing loss. We also incorporated the ReLU activation function between the hidden layers to introduce non-linearity and enable the network to capture complex input-output relationships. These hyperparameters were explored while keeping the number of epochs fixed at 1000. After 1000 epochs, the training loss for the forward modeling network was recorded as $0.0011$, while the test loss was $0.0013$ as shown in Fig. 5a. Once the forward model was adequately trained, we proceeded to freeze its weights to ensure its stability and reliability. This allowed us to use the pre-trained forward model as a fixed component in our architecture. Next, we incorporated the inverse modeling network into the architecture, forming a tandem network. As mentioned above, the main objective of the inverse modeling network was to extract the voltage set for the phase tuning that would produce the desired far-field beam distribution in OPA. By providing the desired beam distribution as input to the trained inverse modeling network, it can generate the corresponding voltage set. The hyperparameters of the inverse modeling network were also optimized using a similar approach as the forward modeling network. The results of the hyperparameter tuning for the forward modeling network and inverse modeling network are summarized in Table 1 below.

**Table 1:** Optimized results of the hyperparameter tuning for the network.

Optimized parameters	Forward modeling network	Inverse modeling network
Number of layers	3	2
Number of nodes	(64)-732-284-610-(350)	(350)-782-949-(64)
Dropout values	0.26	0.19
Learning rates	$4.1\times 10^{-4}$	$0.9\times 10^{-4}$

To ensure that both the generated voltage set and the far-field beam intensity distribution of the entire network closely align with the labels in the training dataset, we introduced an additional structural parameter in the loss function like in our previous study^31^. The loss function comprises 2 components: the voltage set loss ($L_{vs}$) and the far-field beam intensity distribution loss ($L_{id}$). The total loss ($L_{total}$) of the tandem network is computed as a weighted average of these two losses like the formula $L_{total}$=$\alpha$ $L_{vs}$ +(1-$\alpha$)$L_{id}$, where the weight coefficient $\alpha$ is determined through numerous testing. After testing various values, we found that setting $\alpha$ to 0.015 yielded the best results. By incorporating this modified loss function, the network ensures that the generated voltage set and the far-field beam intensity distribution closely resemble the target values in the training dataset. Using the modified loss function, the training for the inverse modeling network was conducted over a total of 1000 epochs and the learning curve can be seen in Fig. 5b. After the training, the network recorded a training loss of 0.0019 and a test loss of 0.0026. As the complexity of the network increased, the training process of the inverse modeling network incorporated the loss from the pre-trained forward modeling network and this led to a slight increase in noise during the learning process. The training process for both the forward modeling network and inverse modeling network took a total of 20 min.

## Experimental results

To validate the performance of our designed network, we utilized the 20 evaluation datasets containing a 64-voltage set and the corresponding far-field beam intensity distribution. As previously mentioned, these datasets were obtained using the HA as a reference.

**Figure 6:**
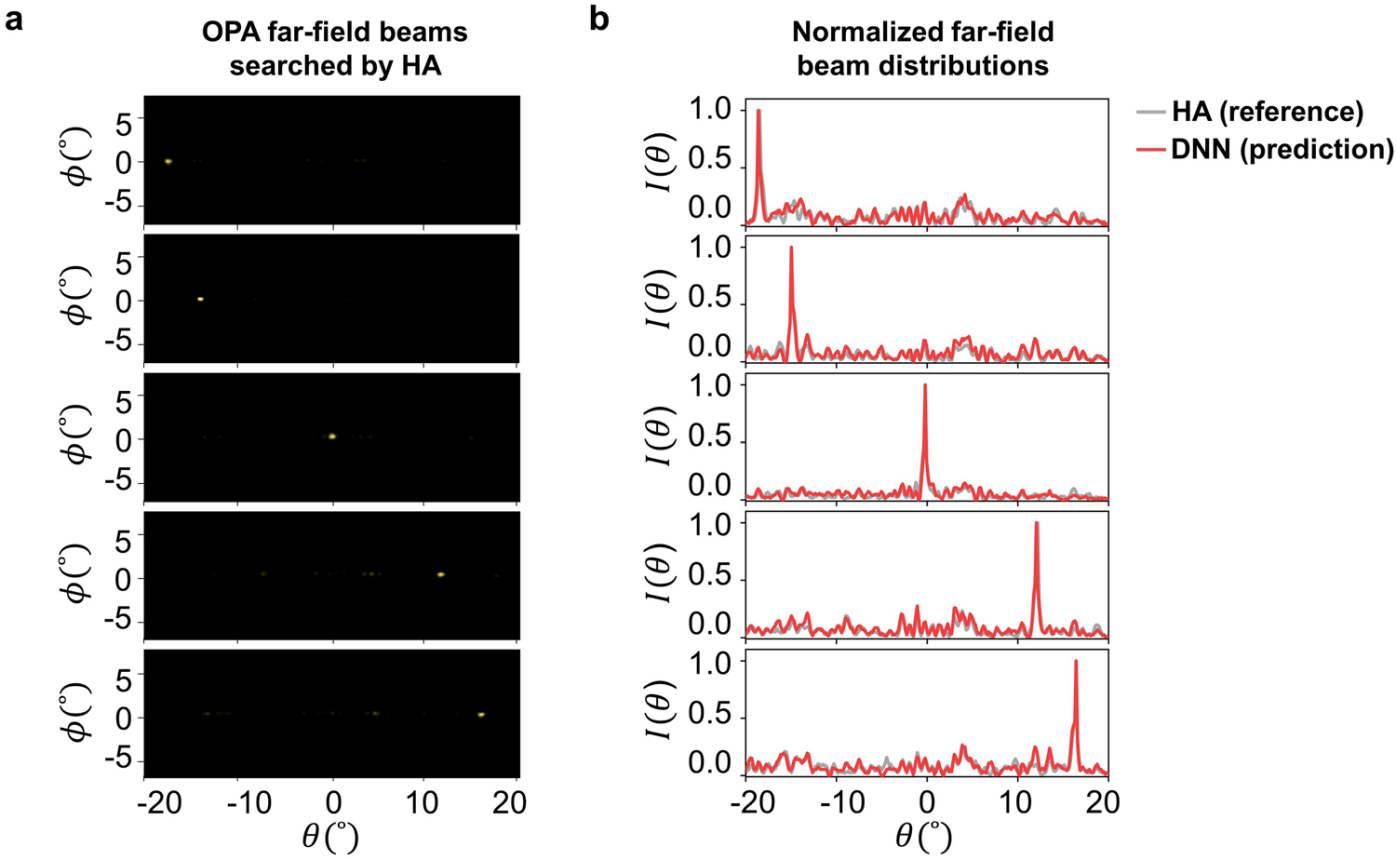
(**a**) Representative OPA far-field beam image results obtained using the hill-climbing algorithm (HA) for 5 different directions. (**b**) Comparison between the normalized far-field beam intensity distribution obtained HA and the normalized predicted beam intensity distribution from the trained network.

**Figure 7:**
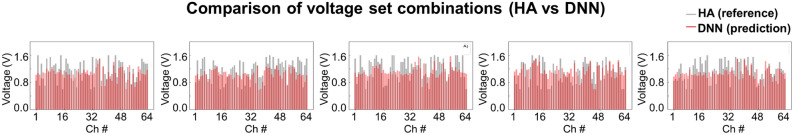
Comparison between the voltage set combination obtained through HA and the voltage set combination extracted from the intermediate layer of the trained network.

Figure 6a shows 5 different representative far-field OPA beam images obtained from the voltage set searched by the HA. These images were captured by an IR camera with the experimental setup described in Fig. 2a. The angular directions of each beam in Fig. 6a are − 18.6$^\circ$, − 13.1$^\circ$, -0.2$^\circ$, 12.1$^\circ$, and 16.4$^\circ$ from the left to the right. The average beam divergence angle for each beam was 0.7$^\circ$ in the transversal direction and 0.6$^\circ$ in the longitudinal direction. The normalized beam intensity distributions, which are individually extracted from the transversal cross-sections of the far-field images in Fig. 6a, are represented by the gray line in Fig. 6b. When these beam intensity distributions are input into our trained DNN model, it generates predicted voltage sets in the intermediate layer, as depicted in Fig. 4. In addition, these voltage sets are subsequently fed into the forward modeling network, resulting in beam intensity distributions represented by the red line in Fig. 6b. Notably, these predicted beam intensity distributions closely match the actual beam intensity distributions obtained through HA, represented by the gray line in Fig. 6b. It suggests that our trained model based on the tandem network successfully captures the complex relationship between the input beam intensity distributions and the corresponding voltage sets, enabling accurate predictions of the resulting beam intensity. Figure 7 presents the comparison between the voltage sets extracted from the intermediate layer of the trained network and the voltage sets obtained through the HA for 5 different far-field beam intensity distributions. Notably, each far-field beam is generated using a unique combination of voltage sets, distinct from the combinations searched by the HA. While this implies the potential of being trapped in local optima due to the non-uniqueness of the voltage sets, it still underscores DNN’s capability to generate a beam similar to the desired high-quality beam found by HA, as evidenced in Fig. 6b.

**Figure 8:**
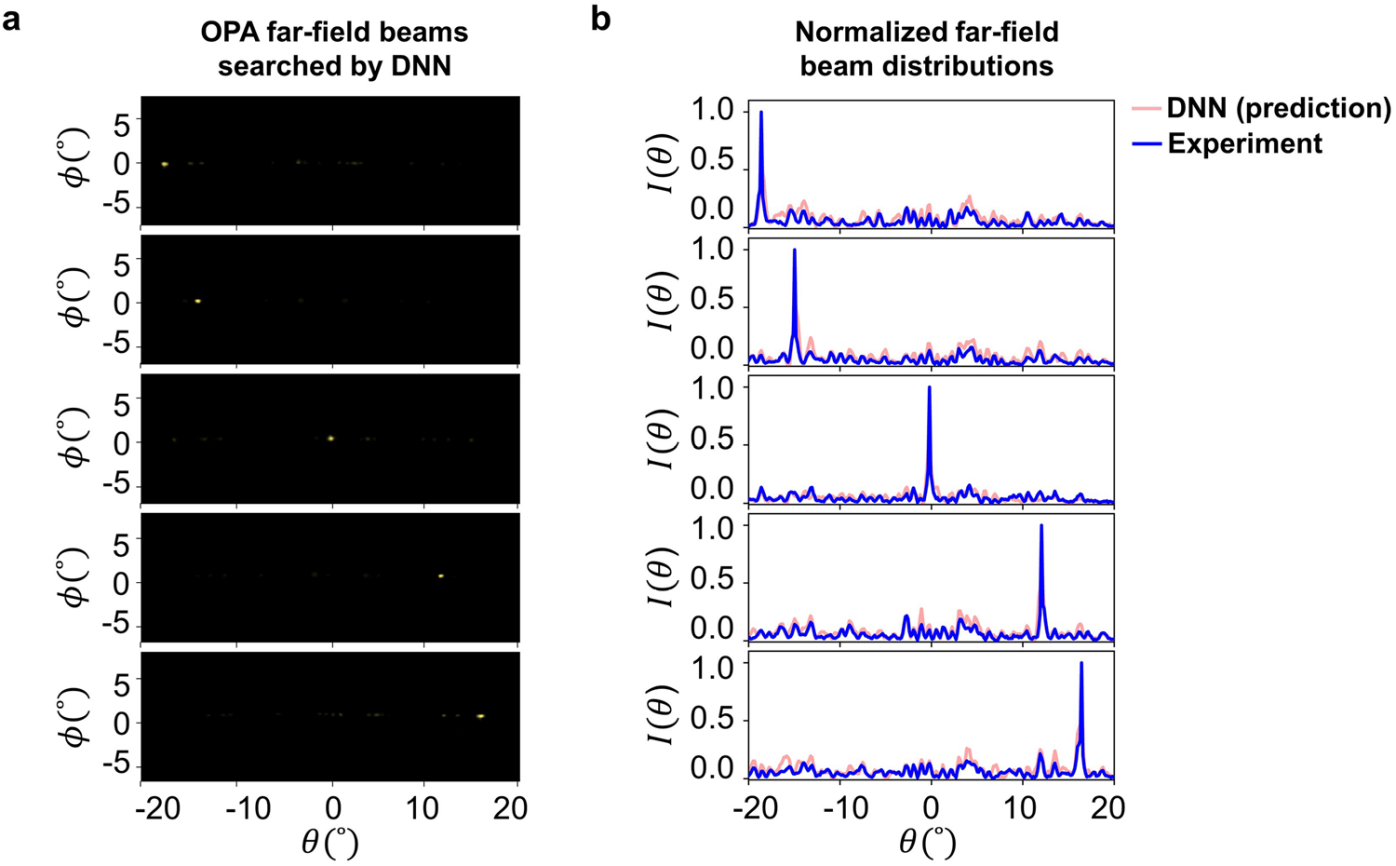
(**a**) OPA far-field beam images using predicted voltage set extracted from the network. (**b**) Comparison of the normalized predicted beam intensity distribution from the trained network and the normalized experimental beam intensity distribution obtained using the predicted voltage set from the network.

The experimental results of the corresponding far-field beams, obtained by inputting these extracted voltage sets into the OPA, are shown in Fig. 8a. Here, we successfully achieved that the predicted voltage set from the trained network closely replicates the desired beam distribution, as evidenced by the obtained results. Figure 8b compares the normalized predicted beam distributions from the network (red line) with the normalized beam distributions (blue line), which are extracted from the transversal cross-sections of the far-field images in Fig. 8a. The normalized experimental beam distributions obtained by using the predicted voltage set from the trained network (blue line) show an average SMSR of 7.05 dB across the five different beam distributions, which is slightly better than the 6.88 dB achieved in the simulated results obtained from the network (red line). In addition, it is worth noting that this level of SMSR is also comparable to the average SMSR of 7.11 dB in the normalized beam distributions obtained using the HA, which is represented by the gray line in Fig. 6b. Including the representative results from the 5 beams, a similar level of consistency was observed in 16 out of the total 20 beams, showing only minor average degradation of 0.06 dB compared to the 7.02 dB average SMSR in the reference beams. For the excluded 4 datasets, 3 exhibited a minor angular shift within 0.3$^\circ$, with over a 2 dB peak intensity degradation compared to each reference case. In just 1 case, the beam prediction significantly deviated from the directional angle of the reference beam. These findings indicate the effectiveness and robustness of our data-gathering method for training the network, as it yields results comparable to those obtained by the HA algorithm.

Nevertheless, it is also important to note that when comparing the far-field images obtained using the HA algorithm in Fig. 6a, there was an average intensity degradation of 0.84 dB in the peak values from all 16 evaluation datasets where beamforming was successfully achieved via the DNN model. This degradation can be attributed to the limited training data, which consisted of only 18,000 selectively sorted samples among $1.9\times 10^{128}$ possible combinations, resulting in a trade-off between accuracy and efficiency. It was evidenced that when an additional 20,000 datasets, collected in the same manner, were fed into the same trained DNN model, we observed an improvement of up to 0.62 dB in the average intensity degradation in the peak values for 16 predicted beams, compared to the reference beams. However, the transition from 18,000 datasets to 38,000 datasets introduced a trade-off, significantly extending the data collection time more than doubling and increasing the training time by approximately 5 times, ultimately leading us to opt for retaining the 18,000 datasets in this study. Despite this trade-off, our DNN-based approach has a great potential for significantly reducing the time required for beam calibration, especially in scenarios with a large number of beam points. In particular, the more OPA beam points the users have to calibrate, the more efficient our DNN approach becomes compared to iterative-based methods like HA, which takes an hour to calibrate only a single beam with 10 iterations in a 64-channel OPA. For instance, while our DNN-based method required approximately 10 h and 20 min for data collection and model training, it is evident that HA is the preferred choice for 10 or fewer beam points due to its shorter time requirements. However, when dealing with 50 or more beam points, our proposed model can demonstrate a time efficiency improvement of over fourfold.

In essence, our approach provides a substantial advantage over HA, as it involves a one-time implementation for dataset collection. Once collected, this dataset can be seamlessly applied to all beam points without additional data collection. This indicates that it is a matter of choosing the most suitable approach, whether iterative algorithms like HA or DNN-based beamforming, depending on the number of beam points that the user intends to search for. For example, in scenarios like point-to-point optical wireless communication, which involve a relatively small number of OPA beam points^4, 5^, HA-based beamforming may be the more suitable choice. In contrast, OPA applications such as 3D image sensing for LiDAR^1–3^, which demands a larger number of resolvable beam points, often numbering in the thousands or more, are expected to benefit significantly from our DNN-based beam calibration. Furthermore, we anticipate that our approach can be applied to OPA designs with higher channel integration beyond 64 or to III/V integrated OPAs equipped with semiconductor optical amplifiers (SOAs), where the phase calibration process is expected to be even more complicated^41^.

Although not within the scope of the present work, it was observed that our structure supports longitudinal beam steering with up to 3W tuning applied to the n-i-n heater, while maintaining the beam intensity distribution in the transversal direction with minimal impact on the SMSR and beam direction angle. This allows for a 2$^\circ$ beam steering in the longitudinal direction using the same predicted voltage set for a specific direction. However, it should be noted that when the power applied exceeds 3W, thermal drift causes variations in the beam intensity distribution, which in turn requires adjusting the combination of voltage sets for effective beamforming. To overcome this challenge and adapt to the 2D beam variation caused by thermal drift, a CNN-based design can be appropriate for training and accommodating these changes^35^. Moreover, in the future work, we expect the application of streamlined optimization algorithms, such as gradient descent-based optimizations and reinforcement learning^42, 43^, which can potentially replace the data preparation method proposed in this study. These approaches are expected to provide solutions to the issue of non-uniqueness in voltage sets and the potential for encountering local optima. These aspects of the problems will be investigated in another study.

## Conclusion

In this study, we have presented the phase calibration methodology for scaled integrated 64-channel OPA based on deep-learning technology. Through meticulous data collection and optimization of the neural network model, we demonstrate its effectiveness in determining the optimal voltage sets for achieving desired beam distributions and experimentally retrieving far-field patterns at several beam direction angles within the steering range of 35$^\circ$. Although there were minor performance degradations compared to the reference beams obtained by the HA method, our experimental results show significant alignment between the DNN-predicted beams and the reference beams. This underscores the potential of our DNN-based approach to streamline time-efficient beam calibration, particularly in scenarios with a multitude of beam points. Furthermore, our approach provides a one-time data collection and training process, eliminating the iterative steps and facilitating intelligent calibration. By enabling time-efficient and precise phase control in scaled integrated OPAs, we expect that our DNN-based approach will provide new possibilities for advancements in optical systems and a wide range of photonic applications.

## Data Availability

The datasets generated and/or analyzed during the current study are available from the corresponding author upon reasonable request.
